# Attitudes and Practices of Dietitians Regarding Gut Microbiota in Health—An Online Survey of the European Federation of the Associations of Dietitians (EFAD)

**DOI:** 10.3390/nu16152452

**Published:** 2024-07-28

**Authors:** Evdokia K. Mitsou, Christina N. Katsagoni, Katarzyna Janiszewska

**Affiliations:** 1 Department of Nutrition and Dietetics, School of Health Science and Education, Harokopio University, 17671 Athens, Greece; 2 Department of Clinical Nutrition, Agia Sofia Children’s Hospital, 11527 Athens, Greece; christina.katsagoni@gmail.com; 3 The European Federation of the Associations of Dietitians (EFAD), Gooimeer 4-15, 1411 DC Naarden, The Netherlands; katarzyna.janiszewska@efad.org

**Keywords:** online survey, dietitians, gut microbiota, probiotics, prebiotics, attitudes, dietetic practice

## Abstract

Explorations of the current attitudes and practices of dietitians regarding the gut microbiota in health are scarce. In this online survey, we assessed the attitudes and practices of dietitians across Europe concerning gut microbiome parameters and the manipulation of the gut microbiota. Pre-graduate dietetic students and other professionals were also invited to participate. The potential interest and preferences of the participants for future educational initiatives about the gut microbiota and the educational resources used were further explored. A total of 179 full responses were recorded (dietitians, n = 155), mainly from the southern and western regions. Most of the participants (>90.0%) believed that probiotics and prebiotics have a place in nutritional practice and that fermented foods with live microbial cultures should be a part of food-based dietary guidelines. A strong belief in the beneficial roles of probiotics and prebiotics in some health situations was also reported among the participants. Most of the dietitians recognised the importance of gut microbiota manipulation and advised the use of probiotics and prebiotics in dietary practice, and they felt quite confident applying the relevant information in their daily practice. Nevertheless, misconceptions were identified, and further guideline-oriented education is necessary. The interest in future e-learning initiatives was high among the participants, and the sources of knowledge, educative formats, and potential areas for further educational efforts were indicated.

## 1. Introduction

The role of gut health is fundamental in maintaining overall human health [1]. The gut microbial ecosystem, termed the gut microbiota, has been repeatedly emphasised as a key player in the interconnections of the gut with other organs, modulating the risk of several chronic diseases [1,2,3,4]. Gut microbiota characteristics, such as composition and functionality, can be influenced by several host or lifestyle factors throughout the life cycle; among these factors, nutrition contributes to shaping gut microbial profiling and metabolic activity [5]. In fact, gut microbiota manipulation through dietary components and proper food choices might offer a novel therapeutic alternative for the treatment of several adverse health outcomes [3]; thus, the use of probiotics and prebiotics as modulators of the gut microbiome is currently under vigorous investigation for the restoration of gastrointestinal and overall health [6].

Understanding the characteristics of the gut microbiota can be significant for both the healthcare provider and the patient, with broad implications for human health [3,7]. Consumers’ interest in probiotics and prebiotics is growing in Europe and worldwide, driving market development and, at the same time, driving a more urgent need for proper public awareness and professional guidance about the parameters and modulation of the gut microbiota [6,8,9,10,11]. In this context, the role of dietitians as healthcare providers is of utmost importance for the translation of scientific evidence about the gut microbiota into practical guidance, thus shaping appropriate consumer choices through dietetic practice [7,12,13].

The practices and attitudes of health care professionals, including dietitians, regarding the use of probiotics and prebiotics have previously been reported [14,15,16,17,18,19]. Nevertheless, explorations focused on the attitudes and practices of dietitians regarding the parameters and modification of the gut microbiota are scarce. In this context, this cross-sectional study aimed to evaluate, through an online survey, the attitudes of qualified dietitians across Europe regarding the beneficial roles of probiotics and prebiotics in specific health conditions (e.g., gastrointestinal disorders, allergies, and metabolic health). Pre-graduate dietetic students and other professionals were also invited to participate. For the dietitians, the current dietetic practices regarding gut microbiota parameters or the use of probiotics and prebiotics in gut microbiota manipulation were additionally assessed. The participants’ potential interest in and preferences for future educational initiatives in the field of the gut microbiota and the educational resources used were further explored. The effect of potential determinants (i.e., European region, age group, educational level, professional background, and perceived/current knowledge) on the tested variables was further analysed.

## 2. Materials and Methods

### 2.1. Subjects

The online survey was an initiative of the European Federation of the Associations of Dietitians (EFAD). Dietitians and pre-graduate dietetic students from around 28 countries with National Dietetic Associations (NDAs) members of the EFAD were invited to participate in the study, and other professionals interested in the field of gut microbiota were also welcomed to take part in the survey. The participants were grouped into one of the four European regions (central and eastern, northern, southern, and western Europe), as previously described [20].

### 2.2. Survey Questionnaire

The survey questions were formulated by the survey project officer (E.K.M.) in collaboration with the Advisory Board members of the EFAD. The survey included 77 questions with 9 sections covering different aspects of gut microbiota, as previously described [20].

The survey included sections about attitudes and beliefs regarding the use of probiotics/prebiotics/fermented foods, aspects of dietary practice regarding gut microbiota parameters (available only for dietitians), sources of relevant knowledge, and interest in future educational initiatives, including areas of interest and e-learning formats. Sections about sociodemographic data (country, age group, educational level, workplace, and years in practice as a dietitian) and the perceived and current knowledge of all the participants in four different fields (gut health and overall health, nutrition as a gut microbiota modulator, probiotics, and prebiotics) were further included in the survey and have been previously reported [20].

For questions regarding the beliefs, attitudes, and practices of the participants, a 5-point Likert scale was applied with answer choices such as “strongly don’t believe”, “don’t believe”, “neutral (don’t know)”, “believe”, and “strongly believe”; “never”, “rarely”, “sometimes”, “often”, and “always”; or “not at all confident”, “not very confident”, “neutral”, “somewhat confident”, and “very confident”. For assessing the interest in future educational initiatives in the gut microbiota field a 5-point Likert scale was applied (“not at all interested”, “not very interested”, “neutral”, “somewhat interested”, and “very interested”) and multiple-choice questions were also included.

The survey was conducted online using the LimeSurvey survey tool (Version 3.5.4+ 180320) (http://www.efadsurveys.eu/index.php/485711?lang=en, accessed on 22 April 2021). The survey communication plan was previously described in detail [20], and the data collection period lasted from 22 April 2021 to 22 July 2021. Participation in the survey was on a completely voluntary basis. The participants were asked to consent before completing the survey. The responses were processed in an anonymised form and were kept confidential, as previously reported [20].

### 2.3. Statistical Analysis

Data are presented as frequencies (n, %) for categorical variables. A cross-tabulation analysis of categorical variables was performed using the chi-squared (X^2^) test, according to the participants’ characteristics (i.e., European region, age group, educational level, professional background, and overall perceived and current knowledge). Correlation analysis (Spearman’s rho) was further applied where applicable. The significance level was set at 5.0% (*p* < 0.05). Statistical analysis was performed using Stata 15.1 [21]. A sample size calculation of at least 380 responses for a representative sample was previously described [20].

## 3. Results

**Figure 1:**
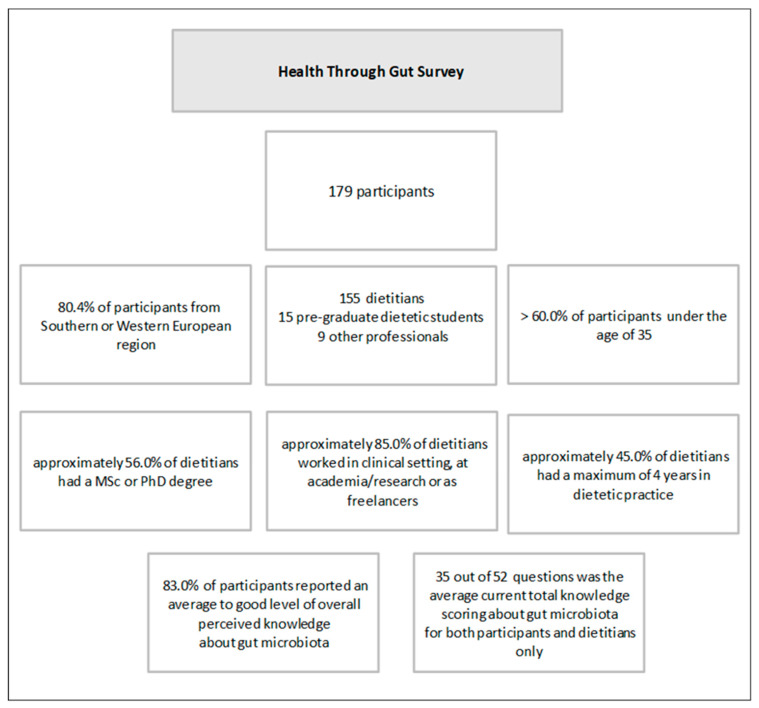
An overview of the Health Through Gut Survey.

Of the one-hundred-seventy-nine participants who provided full responses during the study period, one-hundred-fifty-five were dietitians, fifteen were pre-graduate dietetic students, and nine were other professionals (Figure 1). As previously reported [20], most of the full responses resulted from the southern and western European regions. Most of the participants reported an average to good level of perceived knowledge in the four sections and overall, and significant positive correlations were reported between perceived and current knowledge. The participants’ sociodemographic data and levels of perceived and current knowledge were previously reported in detail [20] and were used for group analysis in this study.

### 3.1. Attitudes towards the Roles of Probiotics, Prebiotics, and Fermented Foods

**Figure 2:**
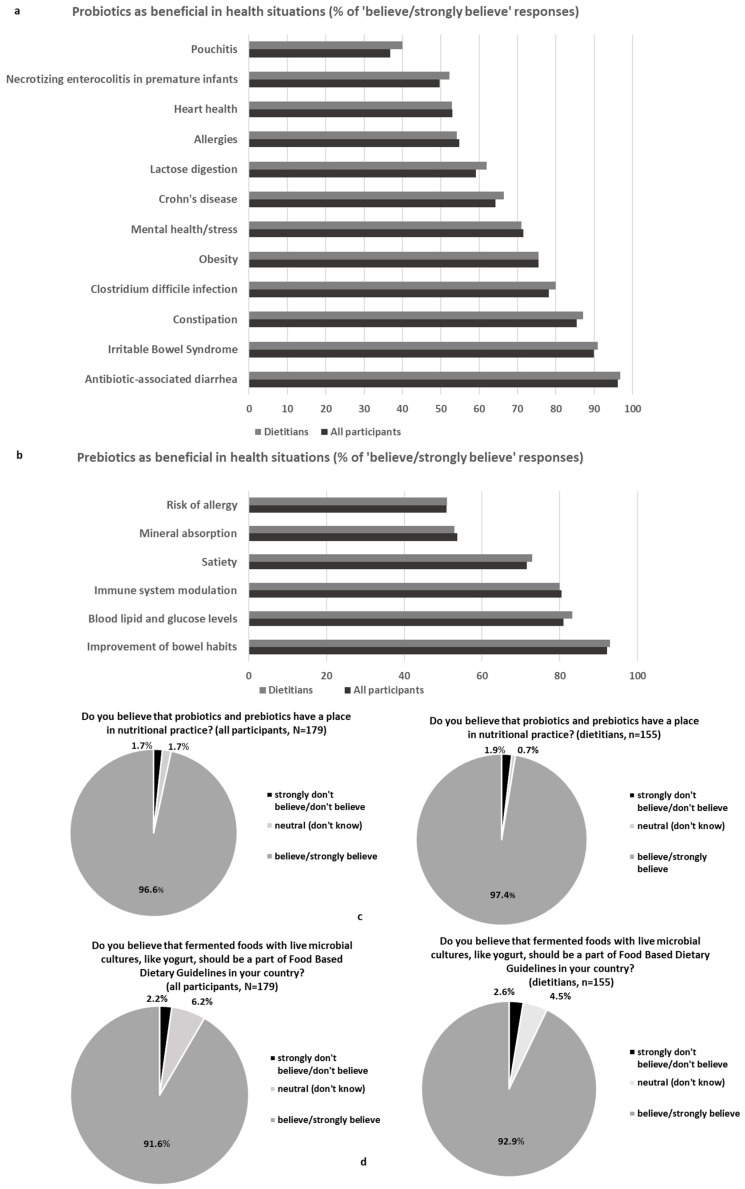
(**a**–**d**) Attitudes of all participants (N = 179) and dietitians (n = 155) in terms of probiotics and prebiotics as beneficial in health situations (**a**,**b**), place of probiotics and prebiotics in nutritional practice (**c**), and fermented foods as part of food-based dietary guidelines (**d**). Values are expressed as a percentage (%) of believe/strongly believe responses (**a**,**b**) or as a percentage (%) of believe/strongly believe, neutral (don’t know), or strongly don’t believe/don’t believe responses (**c**,**d**).

The survey presented to all the participants (N = 179) 5-point Likert questions about their attitudes towards the roles of probiotics, prebiotics, and fermented foods (Figure 2a–d) before moving to the dietetic practice section.

In detail, the participants were asked to report their beliefs about the beneficial roles of probiotics (Figure 2a) and prebiotics (Figure 2b) in specific health conditions. With regard to probiotics, high rates of the beneficial role of probiotics were indicated in the case of antibiotic-associated diarrhoea (AAD) and irritable bowel syndrome (IBS) but also for other health conditions such as obesity or mental health/stress. On the contrary, health situations such as pouchitis or necrotising enterocolitis (NEC) in premature infants had lower rates. Moreover, the rates of “believe/strongly believe” responses overall (N = 179) and in the dietitians’ group (n = 155) were similar (Figure 2a). The participants were also asked to report their beliefs about the beneficial role of prebiotics in specific health conditions (Figure 2b). Similar rates of “believe/strongly believe” responses overall (N = 179) and in the dietitians’ group (n = 155) were also reported. The highest rate of “believe/strongly believe” responses was found for the improvement in the bowel habits (92.0% of the participants/dietitians), while more than 80.0% of the participants/dietitians provided “believe/strongly believe” answers regarding the beneficial role of prebiotics in the improvement in blood lipid and glucose levels. High rates were also reported for immune system modulation (80.0%) and satiety (72.0%), whereas the rates were lower (approximately 50.0%) for mineral absorption and risk of allergy.

Some region and age discrepancies were detected in the use of probiotics for lactose digestion and obesity or the use of prebiotics for mineral absorption and immune system improvement (Supplementary Materials—Tables S1 and S2). For instance, compared to southern or central/eastern Europe, the participants from the northern or western regions were more neutral/in disagreement with the use of probiotics for lactose digestion (overall *p* = 0.015) and obesity (overall *p* = 0.001) or with the use of prebiotics for mineral absorption (overall *p* < 0.001) and immunomodulation (overall *p* = 0.055). Moreover, for all the above health situations, higher rates of belief in the use of probiotics/prebiotics were reported among the younger participants. The dietitians believed more in the use of prebiotics for blood lipids and glucose management compared to students (*p* = 0.045), but those dietitians with more experience were sceptical about the use of probiotics for obesity management (overall *p* = 0.008) and heart health (overall *p* = 0.028) compared to those with fewer years in practice. The participants with poor perceived total knowledge about the gut microbiota believed less in the use of probiotics for lactose digestion, constipation, *Clostridium difficile* infection, pouchitis, or IBS, and in the use of prebiotics for improvement in bowel health, risk of allergy, or satiety compared to those with good/excellent perceived knowledge (*p* for all <0.05). Likewise, the participants with lower current total knowledge scores believed less in the use of probiotics for *C. difficile* infection, Crohn’s disease, pouchitis, or NEC, and in the use of prebiotics for improvement in bowel health, risk of allergy, satiety, or control of blood lipids and glucose compared to those with higher-scoring quartiles (*p* for all <0.05).

In response to the question “Do you believe that probiotics and prebiotics have a place in nutritional practice?”, 97.0% of the dietitians and overall reported they “believe or strongly believe” (Figure 2c). In the case of the workplace (overall *p* = 0.006), the participants in a clinical setting and freelancers exclusively provided “believe or strongly believe” responses (100% of responses), a higher rate compared to those working in community service (87.5%) (vs. clinical setting *p* = 0.010; vs. freelancers, *p* = 0.021) and the “other” option (82.4%) (vs. clinical setting, *p* = 0.008; vs. freelancers, *p* = 0.020). To the question “Do you believe that fermented foods with live microbial cultures, like yoghurt, should be a part of Food-Based Dietary Guidelines in your country?”, 92.0% of the dietitians and overall reported they “believe or strongly believe” (Figure 2d). A comparison among the European regions (overall *p* = 0.029) revealed lower rates of “believe or strongly believe” responses in northern Europe (70.6% of responses) compared to southern (93.9%, *p* = 0.003) or western Europe (93.3%, *p* = 0.039). No further overall significant differences were detected among the tested groups in both questions, and no connections with overall perceived knowledge or total scoring quartiles were observed.

### 3.2. Section: Dietary Practice

**Figure 3:**
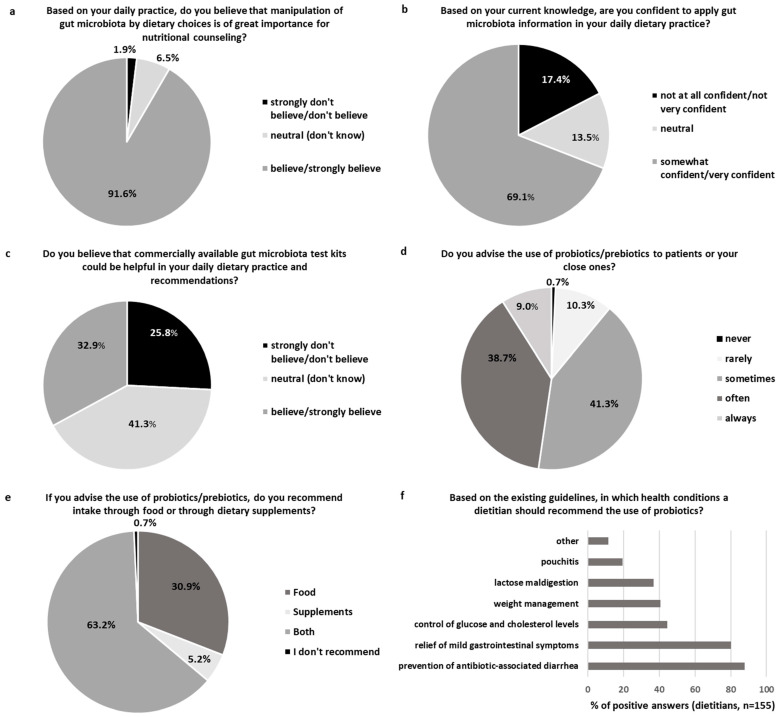
(**a**–**f**) Survey questions about dietetic practice addressed exclusively to dietitians (n = 155); values are expressed as a percentage (%) of different answer options (**a**–**e**) or as a percentage (%) of positive answers per health situation (**f**).

The section about dietary practice was available only for the dietitians (n = 155) (Figure 3a–f). The dietary practice section started with the following question: “Based on your daily practice, do you believe that manipulation of the gut microbiota by dietary choices is of great importance for nutritional counselling?”, with 91.6% of the dietitians reporting they “believe/strongly believe” (Figure 3a). Higher overall perceived knowledge about health maintenance through the gut was positively associated with the rate of “believe/strongly believe” responses to this question (Spearman’s rho 0.219, *p* = 0.006), with poor perceived knowledge associated with a lower rate of “believe/strongly believe”. Those dietitians working as freelancers or in the academic/research field had a greater belief in the importance of gut microbiota manipulation in nutritional counselling (100.0% and 97.4%, respectively) compared to those in community service (80.0%) or other options (66.7%) (*p* for all <0.05), with no further differences in the response rates based on region, age, educational level, or years in practice.

In response to the next question, “Based on your current knowledge, are you confident to apply gut microbiota information in your daily dietary practice?”, 69.1% of the dietitians felt somewhat or very confident about applying relevant gut microbiota information in practice (Figure 3b). Higher overall perceived knowledge was connected to a higher level of confidence (Spearman’s rho 0.424, *p* < 0.001), and poor perceived knowledge was connected to a lower rate of “somewhat/very confident” responses. Further analysis indicated that older dietitians (aged 40–65 years) felt confident applying relevant information at a higher rate (79.5%) compared to their younger colleagues (aged 20–24 years, 45.8%, *p* = 0.019), with no other significant differences among the European regions, educational level, workplaces, or years in practice.

In response to the question “Do you believe that commercially available gut microbiota test kits could be helpful in your daily dietary practice and recommendations?”, 67.1% of the dietitians were neutral or did not believe in the usefulness of gut microbiota test kits in dietary practice (Figure 3c). Nevertheless, the dietitians with excellent overall perceived knowledge had a significantly higher rate of “believe/strongly believe” (81.9% of responses) in the use of gut microbiota test kits compared to the other levels of perceived knowledge (25.0–30.4%; *p* for all <0.05). Moreover, the dietitians aged 30–39 years believed in the usefulness of gut microbiota kits at a higher rate (48.8%) compared to the younger participants (vs. aged 20–24 years: 29.2%, *p* = 0.097; vs. aged 25–29 years:19.6%, *p* = 0.031), with no further differences in the response rates based on region, educational level, workplace, or years in practice.

In response to the question “Do you advise the use of probiotics/prebiotics to patients or your close ones?”, 89.0% of the dietitians answered that they advise at least sometimes the use of probiotics/prebiotics by their patients and close ones (Figure 3d). Higher overall perceived knowledge of the dietitians in health maintenance through the gut positively correlated with the rate of positive responses to this question (Spearman’s rho 0.321, *p* < 0.001), with poor perceived knowledge associated with a lower rate of “sometimes/often/always” responses (62.5% of responses) compared to the other levels of perceived knowledge (88.4–100.0%; *p* for all <0.05). Furthermore, those dietitians with an MSc suggested using probiotics/prebiotics more frequently (93.8%) compared to the first-professional degree holders (vs. pre-BSc, 60.0%, *p* = 0.008; vs. BSc, 82.6%, *p* = 0.029). Among the dietitians who advised the use of probiotics/prebiotics (n = 154), 63.6% recommended intake through both food and dietary supplements, 31.2% through food, and only 5.2% through dietary supplements exclusively (Figure 3e). Further analysis indicated that the dietitians from northern Europe tended to have a higher recommendation rate of probiotics/prebiotics in the form of dietary supplements exclusively (21.4% of the responses) compared to the dietitians from the southern (2.6%, *p* = 0.072) or western areas (4.6%, *p* = 0.069). No further differences according to age groups, educational level, workplace, or years in practice of the participants were detected. Additionally, no significant connection was detected between the overall current level of knowledge regarding health through the gut, as assessed by the total score, and all the above questions regarding the dietary practice in contrast to the observed associations with the participants’ perceived knowledge.

In response to the last question of this section, “Based on existing guidelines, in which health conditions should a dietitian recommend the use of probiotics?”, 87.7% of the dietitians would advise probiotics for AAD and 80.0% for the relief of mild gastrointestinal symptoms based on the guidelines (Figure 3f). Nevertheless, only 19.4% of the dietitians would advise the use of probiotics for pouchitis. Approximately 40.0% of the dietitians would advise the use of probiotics for health conditions such as weight management (40.6%), control of glucose and cholesterol blood levels (44.5%), or lactose maldigestion (36.8%). Further analysis pointed out some region-dependent differences in the question choices, including that (a) all the participating dietitians from western Europe would advise the use of probiotics for AAD compared to 81.8% from the southern (*p* = 0.004) and 85.7% from the northern regions (*p* = 0.016), (b) no participating dietitian from the northern region would advise the use of probiotics for lactose digestion compared to 38.6% from the southern region (*p* = 0.004), 41.7% from central and eastern Europe (*p* = 0.007), and 43.6% from western Europe (*p* = 0.008), and (c) 30.8% of the dietitians from western (*p* = 0.008) and 35.7% from northern Europe (*p* = 0.017) would advise probiotics for pouchitis compared to 11.4% from the southern region. For pouchitis, the dietitians working in a clinical setting would more often advise the use of probiotics (34.6%) compared to freelancers (7.3%, *p* = 0.002) or those in other settings (6.6%, *p* = 0.034), whereas the dietitians aged 25-29 years would less frequently advise the use of probiotics in this health situation (4.3%) compared to the younger (aged 20–24 years: 20.8%, *p* = 0.029) or older ones (aged 30–39 years: 19.5%, *p* = 0.027; aged 40–65 years: 34.1%, *p* < 0.001). For body weight management, the more-experienced dietitians (10–19 years: 23.3%) less frequently recommended probiotics compared to their less-experienced colleagues (vs. 0–4 years: 45.1%, *p* = 0.040; vs. 5–9 years: 51.5%, *p* = 0.021). Likewise, the more-experienced dietitians (10–19 years: 30.0%; 20 years or more: 28.6%) less frequently recommended probiotics for glucose and cholesterol management compared to the dietitians with 0–4 years (47.9%, vs. 10–19 years *p* = 0.097) or 5–9 years of experience (60.6%) (vs. 10–19 years, *p* = 0.015; vs. 20 years or more, *p* = 0.022). No further differences were detected according to the educational level of the participants, and the overall level of perceived or current knowledge had no significant connection with the choice of health conditions in this question.

### 3.3. Section: Sources of Knowledge—Interest in Future Initiatives

The section about the sources of knowledge and the potential interest in future initiatives focusing on the gut microbiota–health interconnections was addressed to all the participants (N = 179) (Supplementary Materials—Figure S1a,d). Based on the survey, the main sources of knowledge about the gut microbiota for the dietitians and overall (>54.0% of responses) were research publications, academic classes, seminars/webinars, and conferences, whereas lower rates were recorded among the participants for media (TV and podcasts) and social media/blogs as sources of information. Other sources of information included books, theses, guidelines, and doctors, among others. Further analysis indicated that (a) the dietitians more often preferred conferences and seminars/webinars compared to the students or other professionals (*p* for all <0.05), whereas the students selected social media/blogs more often (46.6%) compared to the dietitians (13.5%, *p* = 0.001) and, as expected, acquired no information from representatives/companies, compared to 23.9% of the dietitians (*p* = 0.032); (b) the dietitians with a higher educational level (MSc or PhD) or who were in the academic/research field preferred research publications at a significantly higher rate compared to those with basic academic degrees or working elsewhere (*p* for all <0.05); (c) the more-experienced dietitians preferred conferences at a higher rate but chose academic classes/lectures at a lower rate compared to the less-experienced dietitians (*p* for all <0.05); (d) the younger participants (20–24 years) preferred academic classes/lectures at a higher rate compared to the older ones (overall *p* = 0.001); and (e) in western Europe, media (TV and podcasts) were more preferred as a source of knowledge (26.7%) compared to the central and eastern (0.0%, *p* = 0.030), northern (5.9%, *p* = 0.073), or southern regions (7.1%, *p* = 0.001). Furthermore, those participants with good overall perceived knowledge (89.2%) preferred research publications more often compared to those with poor (60.0%, *p* = 0.003) or average perceived knowledge (77.1%, *p* = 0.054); likewise, the participants in the third (88.2%) and fourth (88.1%) quartiles of total scoring preferred research publications more highly compared to the lowest quartile (68.9%, *p* = 0.020 and *p* = 0.033, respectively). In addition, the participants in the third and fourth quartiles of total scoring more often preferred conferences (overall *p* = 0.002) and seminars (overall *p* = 0.056) as the main sources of knowledge compared to the lower-scoring participants, whereas the subjects in the lowest-scoring quartile more often preferred media and TV podcasts (22.2%) compared to the aforementioned groups (vs. third quartile: 0.06%, *p* = 0.020, vs. fourth quartile: 0.07%, *p* = 0.049).

More than 98.0% of the participants and dietitians who took part in the survey were interested in future e-learning initiatives focusing on the gut microbiota, with no overall significant differences in the interest level among the dietitians/pre-graduate dietetic students/other professionals or other tested parameters (regions, age groups, educational level, workplace, and years in practice) (*p* for all >0.05). Educational videos rated the highest (77.0%) for the dietitians and overall as an appealing e-learning format for a future webinar/e-course about health through the gut, followed by video interviews with experts, short articles, infographics, case studies, and slides with voice-overs. The dietitians preferred case studies at a higher rate (61.0%) compared to the students (33.3%, *p* = 0.038) or other professionals (22.2%, *p* = 0.021); those dietitians with a basic degree (86.6%) preferred educational videos at a higher rate compared to those dietitians with a PhD (60.9%, *p* = 0.008) or MSc (75.0%, *p* = 0.092), whereas the dietitians with 5–9 years of practice preferred slides with voice-overs more often compared to the other categories of years in practice (overall *p* = 0.015). Furthermore, the participants with poor perceived knowledge were more interested in short articles (90.0%) compared to those with average (55.4%, *p* = 0.004), good (67.2%, *p* = 0.046), and excellent levels of perceived knowledge (36.4%, *p* = 0.002), with no further significant connections with knowledge level in this section.

Areas of interest such as personalised nutrition through gut microbiota manipulation, dietary patterns and gut microbiota, or the use of probiotics and prebiotics in clinical practice had high rankings (≥85.0%) for the dietitians and all the participants, followed by gut microbiota composition and metabolism in health and disease (78.0%) and gut functions and interconnections with other organs (63.0%). The other areas of interest included connections of the gut microbiota with pregnancy/lactation, autism, or cancer, as suggested by the dietitians/other professionals only. Further analysis indicated that the dietitians were more interested (84.8%) in the use of probiotics and prebiotics in clinical practice compared to the other professionals (44.4%, *p* < 0.001) or pre-graduate dietetic students (73.3%, *p* = 0.100). Moreover, those dietitians with a PhD were less interested in areas such as gut functions and interconnections with other organs or personalised nutrition compared to the BSc/MSc educational groups (*p* for all <0.05), and the most-experienced dietitians were less interested in personalised nutrition compared to those dietitians with four or fewer years in dietetic practice (66.7% vs. 87.3%, *p* = 0.028). Furthermore, the participants in the third quartile of total knowledge were also more interested in the use of probiotics and prebiotics in clinical practice compared to the first quartile (75.6%, *p* = 0.011). For areas of interest such as gut microbiota composition and metabolism or the dietary patterns and gut microbiota, no significant differences were indicated based on the tested parameters (regions, status, age groups, educational level, workplace, years in practice, and perceived and current knowledge).

In response to the question “What factor might have discouraged you from getting trained in the field of health through gut in the past?”, more than 30.0% of the dietitians and all the participants were not discouraged, and approximately 3.0% of the participants reported a lack of interest in the topic. Some discouraging factors were also identified, including financial expenses for travel and conference fees (approximately 26.0% of the participants/dietitians) and the perceived complexity of the relevant information regarding the terminology, functions, and application of the gut microbiota in dietetics (approximately 14.0% of the participants/dietitians). The other factors included the lack of time and lack of evidence-based courses specific to dietary practice. Further analysis indicated no significant differences in this question based on the tested parameters (status, regions, age groups, educational level, workplace, years in practice, and perceived and current knowledge).

Finally, in response to the last question of the section, “How much money on average do you spend annually for nutrition training?”, 64.0% of the dietitians spent less than EUR 500.00 annually for nutrition training, with the younger participants usually reporting less than EUR 100.00 annual expenses. Lastly, in this study, proportionally fewer dietitians did not usually undertake nutritional training (7.0%) compared to the other professionals (44.4%, *p* = 0.009) and pre-graduate dietetic students (20.0%, *p* = 0.064). Moreover, those participants with good perceived overall knowledge participated more often and spent more money on nutrition training compared to the other participants (overall *p* = 0.068).

## 4. Discussion

In this cross-sectional study, we assessed the attitudes and practices of dietitians around Europe in the field of the gut microbiota in health through an online survey. Pre-graduate dietetic students and other professionals were also invited to participate. The data were further analysed according to the participants’ characteristics. A total of 179 full responses were recorded (dietitians, n = 155). Most of the participants (>90.0%) believed that probiotics and prebiotics have a place in nutritional practice and that fermented foods with live microbial cultures should be a part of food-based dietary guidelines. A strong belief in the beneficial roles of probiotics and prebiotics in specific health conditions was also reported among the participants. Most of the dietitians recognised the importance of gut microbiota manipulation in dietary practice and advised the use of probiotics and prebiotics, while they felt quite confident in applying relevant information in their daily practice. Nevertheless, misconceptions and discrepancies were identified, and the need for further guideline-oriented education was highlighted. The interest in future e-learning initiatives was high among the participants, where sources of knowledge, educative formats, and potential areas for further educational efforts were indicated.

Most of the participants (97.0%) in this study believed that probiotics and prebiotics have a place in nutritional practice, rating them higher compared to previous data regarding probiotics (83.6%) [17]. High rates of belief in the beneficial role of probiotics were indicated in the cases of AAD and IBS, as the previously reported data ranged from 63.3% to 95.0% for AAD and 58.5% to 95.0% for IBS [15,17,18], but also in the cases of other health conditions such as obesity or mental health/stress. On the contrary, lower rates were reported in health situations such as NEC in premature infants or pouchitis, in accordance with the previous results indicating rates of 30.0% for pouchitis and 40.0% for NEC [18], despite the evidence-based indications and conditional recommendations available [22,23,24]. Furthermore, poor knowledge about the available therapeutic choices possibly resulted in lower belief rates about the beneficial role of probiotics in some health conditions, e.g., in the case of *C. difficile* infection, where the conditional use of probiotics [24] or faecal microbiota transplantation [20] could be applicable in clinical practice. The participants were further asked to report their beliefs about the beneficial role of prebiotics in specific health situations. The highest rate of “believe/strongly believe” responses (92.0% of the participants/dietitians) was reported in the case of bowel habit improvement, in accordance with the authorised EU health claim for chicory inulin in the maintenance of normal defecation by increasing stool frequency [25]. Nevertheless, more than 80.0% of the participants/dietitians believed or strongly believed in the beneficial role of prebiotics to improve blood lipid and glucose levels; these are some of the health benefits accepted by the EFSA and US Food and Drug Administration (FDA) for dietary fibres, but not specifically for prebiotics [26,27]. In addition to improvement in blood lipid and glucose levels, high rates were also reported for immune system modulation (80.0%) and satiety (72.0%), whereas they were lower (approximately 50.0%) for mineral absorption and risk of allergy; these are health indications where specific prebiotics could be considered in dietetic practice based on the available randomised controllable data [28], although authorised health claims or strong recommendations are not yet available.

Most of the participants believed that fermented foods with live microbial cultures should be a part of food-based dietary guidelines. Among fermented products, the consumption of yoghurt and other fermented milk products has been extensively associated with beneficial effects on gastrointestinal, cardiovascular, and metabolic health [29,30,31]. In addition, the consumption of the live bacterial cultures in yoghurt has a health claim approved by the European Food and Safety Authority (EFSA) due to the improvement in the lactose digestion in the gut [32]. Nevertheless, only a few European national nutrition guidelines recommend the consumption of yoghurt as part of a healthy diet based on the potential health benefits of its active bacterial content per se [33]. Thus, live yoghurt as a highly bioactive, nutrient-dense, safe, and affordable food can be encouraged as part of a national food-based dietary guideline [29], and other fermented milk dairy products with live bacteria can also be considered for dietary strategies to improve health [31]. In this context, the role of fermented foods with live microbial cultures can be further emphasised, alongside probiotic consumption, as a means of including higher levels of safe microbes with positive health implications in the daily diet [34,35]. Nevertheless, further scientific efforts are necessary to translate the evidence-based knowledge about fermented foods into standard dietetics practice [7].

In a continuum, this cross-sectional study shed some light on the attitudes regarding the dietetic practice of nutrition experts about the gut microbiota. Most of the dietitians (>90.0%) participating in the survey recognised that gut microbiota manipulation by dietary choices is of great importance for nutritional counselling, where 69.1% of the dietitians felt confident about applying the relevant information in their daily practice. The overall perceived knowledge of the dietitians about the topic was positively associated with both parameters, and the workplace or age of the participants had further implications. In the qualitative study by Williams et al., dietitians in Australia recognised the importance of the dietary manipulation of the gut microbiota; nevertheless, the reported confidence in this practice was more limited, partially due to translatable research restrictions [7]. In our study, 67.1% of the dietitians did not advocate for the usefulness of commercially available gut microbiota test kits in dietary practice. In line with this, the available scientific documentation has not supported to date the use of commercial gut microbiota test kits for dietetic practice, as advised by the Gut Microbiota for Health (GMfH) Expert Panel of the British Society of Gastroenterology (available at https://www.bda.uk.com/resource/commercial-gut-microbiome-testing.html, accessed on 15 March 2024). Nevertheless, those dietitians with excellent overall perceived knowledge were more prone to test kit application, indicating a misconception in the field.

Most of the dietitians advised the use of probiotics and prebiotics in clinical practice and for their close ones. This is in line with the previous data on healthcare professionals [14,36,37], although lower rates in dietitians have also been noticed [17]. Factors such as the overall perceived knowledge of the dietitians about the topic or a higher educational level were positively associated with the likelihood of probiotics/prebiotics recommendation in daily practice in this study. Other variables, such as the reported level of training in probiotics [17], knowledge about the safety and efficacy of probiotics [37], concerns about probiotics use, including cost and lack of documentation [18,36], or sociocultural background and the previous experiences of health care providers [37], have also been linked to the likelihood of probiotic recommendation in the past. The tendency for a higher recommendation rate of probiotics and prebiotics exclusively in the form of dietary supplements by dietitians from northern Europe compared to the southern or western regions may reflect different trends in the probiotic product markets around Europe. For instance, Italy, Germany, and France together represented 51.3% of the 2022 western and eastern European sales of probiotic supplements, whereas Spain was the leading EU market for the functional/fortified probiotic yoghurts and sour milk product markets spread across eastern and western Europe (available at https://www.ipaeurope.org/legal-framework/market-data/, accessed on 15 March 2024). Furthermore, the sociocultural variations in the dietary habits, food availability, and consumer preferences among the European regions might also explain the discrepancies in the dietary practice choices [38].

Based on the existing guidelines, 87.7% of the dietitians would advise probiotics for AAD and 80.0% for the relief of mild gastrointestinal symptoms. This fact agrees with the previous data from dietitians and other health professionals [14,17,18,37,39,40] and can be characterised as a good practice based on the available guidelines for children and adults [24,41,42]. In contrast, around 40.0% of the dietitians in our study would advise the use of probiotics for health conditions with no clinical guidelines available yet, such as weight management or control of glucose and cholesterol blood levels [43]; in this case, the years of experience as a dietitian was a crucial factor in the proper recommendation of probiotics. Additionally, 36.3% of the dietitians would advise the use of probiotics for lactose maldigestion, a health condition with an EFSA health claim for live starter cultures of yoghurt but not for a specific probiotic strain [32]. Further analysis pointed out some region-dependent differences in the responses. Strikingly, no participating dietitian from the northern region would advise the use of probiotics for lactose digestion compared to approximately 40.0% of the dietitians from the other European regions. Lactose tolerance is exceptionally widespread in northern European countries, reaching levels of 74% and 82% in Sweden and Finland, respectively [44], whereas around 60% of the adult population in central Europe is lactose-tolerant, compared to only 20% in southern Europe [45]. Thus, our data may depict discrepancies in lactose tolerance across Europe and the subsequent need for dietetic management. Lastly, only 19.4% of the dietitians in the current study would advise the use of probiotics for pouchitis, a complication of surgically treated ulcerative colitis with evidence-based indications and conditional guidelines for probiotics use [22,24]. Furthermore, more dietitians from western and northern Europe would advise probiotics for pouchitis compared to their colleagues from the southern region. This observation may be related to the documented higher incidence of inflammatory bowel disease (IBD) in northern compared to southern Europe [46,47] or to the recorded higher incidence of clinical occupational background in the study participants from the northern compared to the southern region, thus contributing to the different exposure levels of the dietitians to relevant patient cases. In addition, the dietitians working in a clinical setting more often advised the use of probiotics for pouchitis, probably due to their greater clinical experience with similar cases, whereas the younger dietitians would less frequently advise the use of probiotics in this health situation, probably because of a shortage of relevant knowledge or experience. Although no specific question regarding probiotic strain or dosage selection based on the health condition was included in this study, our data pointed out as previously [39] the need to establish and communicate clear and consistent evidence-based guidelines among healthcare professionals for solid practice choices.

This work further addressed the parameters regarding the sources of knowledge and the potential interest in future initiatives on health through the gut. In the past, various sources of information about probiotics or the gut microbiota have been noted [7,14,17,40]. This study highlighted the dynamics of the different sources of knowledge based on the participants’ characteristics and, thus, the need to address potential educational gaps in the field through various means and communication channels. For instance, the younger participants aged 20–24 years more often reported academic classes/lectures as sources of information about health through the gut, whereas the pre-graduate dietetic students more often preferred social media/blogs for acquiring the relevant information. Thus, the further incorporation of gut microbiota-oriented classes into academic curricula and exploitation of communication networks for educational purposes [48] can be future initiatives for these groups. Furthermore, research publications or conferences and seminars/webinars might better serve as future educational alternatives for dietitians with a higher educational level or greater experience and in general for participants with a greater level of knowledge about the gut microbiota. On the other hand, the participants with low current scores on gut microbiota more frequently preferred TV, media, and podcasts as sources of knowledge; this raises a concern about the overall quality and reliability of the available videos and emphasises the need to protect audiences from misinformation about the gut microbiome field via popular media [49].

The interest in future e-learning initiatives was high among the participants in this study, with educational videos rated first in the participants’ preferences among several alternatives, emphasising their role as a highly effective educational tool [50]. As expected, case studies were more appealing for the dietitians compared to the other groups in this study as a means to improve their practice competence and skills. Short articles were preferable among those participants with poor perceived overall knowledge; although press sources, like short articles, have boosted the popularity and digestibility of gut microbiome information over the years, attention should be paid to their limited contribution to communicating evidence-based science among audiences [51].

Areas of interest such as personalised nutrition through gut microbiota manipulation, connections of dietary patterns with gut microbiota, or the use of probiotics and prebiotics in clinical practice had high rankings (≥85.0%) for the dietitians and all the participants. The dietitians were more interested in the use of probiotics and prebiotics in clinical practice compared to the other professionals, reflecting their potential interest in acquiring further knowledge about evidence-based recommendations for gut microbiota manipulation. Personalised nutrition through gut microbiota manipulation is a rising scientific field with current advances [52], but the need for further documentation could justify the scepticism of dietitians with a higher education level or greater experience towards relevant education initiatives. Lastly, parameters about the cost of training, the perceived complexity in the application of gut microbiota information in dietetics, or the lack of evidence-based courses specific to dietary practice were reported previously [7] and should be considered for future initiatives.

## 5. Conclusions

In conclusion, this online survey provided feedback about the attitudes and practices of dietitians across Europe in the field of the gut microbiota according to the participants’ characteristics. Feedback from pre-graduate dietetic students and other professionals was also included. Considering the previously reported limitations of this online survey regarding participation imbalances among European regions and the level of representativeness of the queried population [20], further scientific efforts are necessary before extrapolating these results to the European level. Regarding the high interest of the participants in educational initiatives, the analysis of the current data can fuel future efforts to develop high-quality, life-long learning materials about the gut microbiota focused on dietitians’ need for evidence-based guidance in their daily practice. The implementation of surveys among healthcare professionals regarding other emerging fields in the study of the gut microbiota (e.g., postbiotics, live biotherapeutic products, next-generation probiotics, and candidate prebiotics) will further advance aspects of clinical practice in the future.

## Data Availability

The data presented in this study are available upon reasonable request from the corresponding author. The data are not publicly available due to legal restrictions by the EFAD.

## Supplementary Materials

The following supporting information can be downloaded at https://www.mdpi.com/article/10.3390/nu16152452/s1, Figure S1: (a–d) Survey questions addressed to all participants (N = 179) and dietitians (n = 155) about sources of knowledge and interest in future initiatives regarding health through the gut; values are expressed as the percentage (%) of different answer options; Table S1: Effect of potential determinants (i.e., European region, age group, educational level, professional background, and perceived/current knowledge) on the attitudes of all participants in terms of probiotics as beneficial in health situations (full responses, N = 179) *; Table S2: Effect of potential determinants (i.e., European region, age group, educational level, professional background, and perceived/current knowledge) on the attitudes of all participants in terms of prebiotics as beneficial in health situations, and place of probiotics and prebiotics in nutritional practice and fermented foods as part of food-based dietary guidelines (full responses, N = 179) *.
