# Supplementary material for: Attitudes and Practices of Dietitians Regarding Gut Microbiota in Health—An Online Survey of the European Federation of the Associations of Dietitians (EFAD)
Source: Nutrients. 2024 Jul 28;16(15):2452. doi: 10.3390/nu16152452 (PMC11314052; doi:10.3390/nu16152452)
Supplement: Supplementary file 1 [file nutrients-16-02452-s001.zip › nutrients-3086165-supplementary.pdf]

Table S1: Effect of potential determinants (i.e. European region, age group, educational level, professional background, perceived/current knowledge) on the attitudes of all participants in terms of probiotics as beneficial in health situations (full responses, N=179)*

Subjects' characteri-stics	Variable categories	Lactose digestion	Consti-pation	AAD	*Clostridium difficile* in-fection	Crohn's disease	Pouchitis	NEC	IBS	Allergies	Obesity	Heart health	Mental Health
European region†	Central and Eastern (n=14)	3/0/11	1/1/12	0/0/14	0/1/13	1/4/9	0/8/6	1/5/8	0/1/13	1/3/10	1/1/12	2/4/8	0/2/12
	Northern (n=17)	7/5/5	1/4/12	0/0/17	1/2/14	3/5/9	1/9/7	1/10/6	0/0/17	4/6/7	1/8/8	1/8/8	3/5/9
	Southern (n=99)	12/23/64	2/9/88	2/5/92	2/22/75	5/27/67	5/62/32	4/42/53	3/8/88	10/35/54	5/11/83	9/34/56	5/21/73
	Western (n=45)	9/13/23	4/3/38	0/0/45	0/11/34	4/14/27	1/24/20	3/22/20	2/4/39	2/20/23	0/17/28	3/22/20	4/10/31
	**overall *P***	**0.015**	0.238	0.470	0.463	0.665	0.760	0.776	0.807	0.252	**0.001**	0.639	0.344
Age group (years)	20-24 (n=33)	7/8/18	1/5/27	1/4/28	1/6/26	3/9/21	1/23/9	1/16/16	2/3/28	3/13/17	1/4/28	3/9/21	0/4/29
	25-29 (n=51)	4/8/39	1/5/45	1/1/49	1/10/40	2/13/36	2/33/16	4/23/24	1/5/45	8/15/28	5/6/40	5/18/28	3/12/36
	30-34 (n=27)	4/9/14	1/1/25	0/0/27	0/6/21	3/6/18	1/15/11	0/13/14	0/1/26	2/7/18	1/3/23	3/7/17	3/5/19
	35-39 (n=14)	5/5/14	1/2/44	0/0/14	0/1/13	0/4/10	0/9/5	2/4/8	0/0/14	1/4/9	0/5/9	1/6/7	0/4/10
	40-44 (n=16)	4/3/9	2/1/13	0/0/16	0/5/11	2/4/10	0/8/8	1/5/10	1/1/14	1/7/8	0/6/10	2/7/7	2/2/12
	45-49 (n=16)	1/8/7	1/2/13	0/0/16	0/5/11	1/5/10	1/9/6	0/8/8	1/3/12	0/8/8	0/4/12	0/9/7	1/5/10
	50-54 (n=17)	5/1/11	1/2/14	0/0/17	0/2/15	2/7/8	1/7/9	1/9/7	0/0/17	2/8/7	0/7/10	1/11/5	2/5/10
	55-59 (n=4)	1/0/3	0/0/4	0/0/4	1/1/2	0/3/1	1/2/1	1/2/1	0/0/4	0/2/2	0/1/3	0/1/3	1/1/2
	60-65 (n=1)	0/0/1	0/0/1	0/0/1	0/0/1	0/0/1	0/0/1	0/0/1	0/0/1	0/0/1	0/1/0	0/1/0	0/1/0
	**overall *P***	**0.039**	0.963	0.445	0.201	0.789	0.630	0.714	0.731	0.831	0.075	0.518	0.398
Status	Dietitian (n=155)	25/34/96	8/12/135	2/3/150	2/29/124	12/40/103	6/87/62	8/66/81	5/9/141	15/56/84	6/32/117	12/61/82	10/35/110
	Other professional (n=9)	2/4/3	0/3/6	0/0/9	1/3/5	0/3/6	1/6/2	1/4/4	0/2/7	1/2/6	1/1/7	1/3/5	1/3/9

	Pre-graduate dietetic student (n=15)	4/4/7	0/3/12	0/2/13	0/4/11	1/8/6	0/13/2	1/10/4	0/2/13	1/6/8	0/4/11	2/5/8	1/1/13
	**overall *P***	0.339	0.065	0.131	0.130	0.205	0.125	0.379	0.293	0.918	0.653	0.938	0.516
Educational level (dietitians) †	Dietitian, BTS (n=4)	0/1/3	1/1/2	0/0/4	0/3/1	0/2/2	0/4/0	1/2/1	1/0/3	0/3/1	0/0/4	0/2/2	0/2/2
	Dietitian, pre-BSc (n=5)	0/1/4	0/0/5	0/0/5	0/2/3	0/3/2	0/5/0	0/1/4	0/0/5	0/2/3	1/1/3	1/2/2	1/2/2
	Dietitian, BSc (n=58)	8/14/36	1/5/52	1/2/55	1/9/48	4/18/36	3/31/24	3/29/26	2/4/52	4/24/30	2/10/46	3/24/31	3/8/47
	Dietitian, MSc (n=65)	14/13/38	4/6/55	1/1/63	1/10/54	7/11/47	2/37/26	4/24/37	2/3/60	9/20/36	3/14/48	6/25/34	4/18/43
	Dietitian, PhD (n=23)	3/5/15	2/0/21	0/0/23	0/5/18	1/6/16	1/10/12	0/10/13	0/2/21	2/7/14	0/7/16	2/8/13	2/5/16
	**overall *P***	0.877	0.256	0.983	0.746	0.354	0.340	0.361	0.423	0.612	0.485	0.960	0.365
Workplace †	Clinical setting (n=52)	12/11/29	3/6/43	1/0/51	1/12/39	6/17/29	3/27/22	3/24/25	1/1/50	7/19/26	2/16/24	4/24/24	3/13/36
	Community service (n=8)	3/2/3	0/1/7	0/0/8	1/1/6	0/2/6	1/5/2	1/4/3	0/2/6	2/2/4	1/1/6	1/2/5	2/1/5
	Industry (n=6)	0/2/4	1/0/5	0/0/6	0/0/6	0/1/5	0/3/3	0/2/4	0/0/6	1/2/3	1/0/5	1/1/4	0/1/5
	Academia/ Research (n=39)	5/9/25	3/3/33	0/1/37	1/5/33	4/8/27	2/20/17	1/18/20	1/6/32	4/13/22	2/10/27	4/16/19	0/1/5
	Freelancer (n=42)	5/12/25	0/3/39	0/1/41	0/10/32	1/11/30	1/26/15	1/18/23	1/1/40	1/16/25	1/3/38	2/16/24	4/9/26
	Other (n=17)	2/2/13	1/2/14	0/1/16	0/4/13	1/4/12	0/12/5	3/4/10	2/1/14	1/6/10	0/3/14	1/5/11	0/5/12
	**overall *P***	0.515	0.761	0.913	0.432	0.701	0.841	0.452	0.060	0.775	0.117	0.856	0.643
Years in practice as a dietitian†	0 – 4 (n=71)	11/15/45	2/7/62	2/3/66	2/14/55	6/15/50	5/44/22	5/32/34	3/6/62	10/25/36	4/9/58	7/23/41	4/14/53
	5 – 9 (n=33)	5/9/19	2/2/29	0/0/33	0/8/25	4/7/22	0/21/12	0/15/18	1/0/32	1/11/21	1/3/29	1/10/22	1/6/26

	10 – 19 (n=30)	7/7/16	3/2/25	0/0/30	0/3/27	1/10/19	1/15/14	3/10/17	1/1/28	3/11/16	1/12/17	4/14/12	3/8/19
	20 or more (n=21)	2/3/16	1/1/19	0/0/21	0/4/17	1/8/12	0/7/14	0/9/12	0/2/19	1/9/11	0/8/13	0/14/7	2/7/12
	**overall *P***	0.706	0.800	0.411	0.587	0.531	0.052	0.434	0.577	0.605	**0.008**	**0.028**	0.591
Total perceived knowledge	Poor (n=20)	2/11/7	0/5/15	0/2/18	0/11/9	2/7/11	0/17/3	1/12/7	3/2/15	2/13/5	0/7/13	0/10/10	1/6/13
	Average (n=83)	18/21/44	2/10/71	1/2/80	2/14/67	4/27/52	2/57/24	7/37/39	1/4/78	8/23/52	5/14/64	9/31/43	5/20/58
	Good (n=65)	9/10/46	4/3/58	1/1/63	1/11/53	5/16/44	5/31/29	2/28/35	1/7/57	5/26/34	2/13/50	5/24/36	5/10/50
	Excellent (n=11)	2/0/9	2/0/9	0/0/11	0/0/11	2/1/8	0/1/10	0/3/8	0/0/11	2/2/7	0/3/8	1/4/6	1/3/1
	**overall *P***	**0.003**	**0.021**	0.545	**0.003**	0.443	**<0.001**	0.341	**0.016**	**0.042**	0.489	0.781	0.804
Total current Knowledge	1st quartile (n=45)	7/16/22	0/8/37	1/2/42	1/19/25	3/20/22	0/36/9	1/28/16	3/3/39	4/19/22	2/11/32	3/19/23	4/10/31
	2nd quartile (n=41)	7/9/25	1/3/37	0/2/39	0/9/32	1/11/29	0/29/12	5/20/16	1/4/36	6/14/21	1/5/35	3/12/26	1/8/32
	3rd quartile (n=51)	10/8/33	1/4/46	0/1/50	1/6/44	4/15/32	3/23/25	4/20/27	0/3/48	5/19/27	2/8/41	4/21/26	3/13/35
	4th quartile (n=42)	7/9/26	6/3/33	1/0/41	1/2/39	5/5/32	4/18/20	0/12/30	1/3/38	2/12/28	2/13/27	5/17/20	4/8/30
	**overall *P***	0.467	**0.012**	0.600	**0.001**	**0.034**	**0.001**	**0.003**	0.600	0.564	0.386	0.784	0.822

* Values are expressed as counts (n) of cases of "strongly don't believe - don't believe"/ "neutral (don't know)" / "believe - strongly believe" for each variable category for the different health conditions; Numbers in parenthesis are counts (n) of cases per variable category and overall p depicts crosstabulation (chi-squared) analysis; †For European region n=175, for educational level and years in practice as a dietitian n=153, for workplace n=161; AAD: antibiotic-associated diarrhoea; NEC: necrotizing enterocolitis; IBS: irritable bowel syndrome

Table S2: Effect of potential determinants (i.e. European region, age group, educational level, professional background, perceived/current knowledge) on the attitudes of all participants in terms of prebiotics as beneficial in health situations, place of probiotics and prebiotics in nutritional practice and fermented foods as part of food-based dietary guidelines (full responses, N=179)*

Subjects' characteristics	Variable categories	Improvement of bowel habits	Mineral Absorption	Immune System modulation	Risk of allergy	Satiety	Blood Lipids and Glucose levels	Place of probiotics/ prebiotics in clinical practice	Fermented foods as part of FBDGs
European region†	Central and Eastern (n=14)	0/014	1/5/8	0/3/11	2/2/10	1/2/11	0/1/13	0/0/14	1/0/13
	Northern (n=17)	0/2/15	5/8/4	2/3/12	2/10/5	0/5/12	0/2/15	1/0/16	1/4/12
	Southern (n=99)	0/9/90	5/27/67	1/16/82	7/39/53	4/28/67	4/19/76	2/1/96	2/4/93
	Western (n=45)	0/3/42	4/26/15	0/10/35	2/24/19	1/10/34	2/6/37	0/2/43	0/3/42
	**overall *P***	0.612	**<0.001**	0.055	0.105	0.794	0.732	0.470	**0.029**
Age group (years)	20-24 (n=33)	0/3/30	4/7/22	1/4/28	3/11/19	0/10/23	2/6/25	1/0/32	0/3/30
	25-29 (n=51)	0/3/48	1/18/32	0/11/40	3/22/26	2/13/36	2/10/39	1/1/49	3/2/46
	30-34 (n=27)	0/3/24	2/9/16	0/1/26	2/10/15	2/2/23	1/1/25	0/0/27	0/2/25
	35-39 (n=14)	0/2/12	1/5/8	0/1/13	2/7/5	1/3/10	0/1/13	0/0/14	0/0/14
	40-44 (n=16)	0/2/14	1/7/8	0/6/10	0/7/9	0/5/11	1/3/12	0/1/15	0/0/16
	45-49 (n=16)	0/0/16	1/10/5	0/3/13	0/6/10	1/5/10	0/3/13	0/0/16	0/3/13
	50-54 (n=17)	0/1/16	4/8/5	1/5/11	2/9/6	0/5/12	0/3/14	1/1/15	1/1/15
	55-59 (n=4)	0/0/4	1/3/0	1/0/3	1/2/1	0/2/2	0/1/3	0/0/4	0/0/4
	60-65 (n=1)	0/0/1	0/1/0	0/1/0	0/1/0	0/0/1	0/0/1	0/0/1	0/0/1
	**overall *P***	0.865	0.059	**0.005**	0.767	0.765	0.937	0.909	0.579
Status	Dietitian (n=155)	0/11/144	13/60/82	3/28/124	9/67/79	6/36/113	3/23/129	3/1/151	4/7/144

	Other professional (n=9)	0/1/8	0/4/5	0/2/7	2/2/5	0/3/6	1/3/5	0/1/8	0/2/7
	Pre-gradu-ate die-tetic student (n=15)	0/2/13	2/4/9	0/2/13	2/6/7	0/6/9	2/2/11	0/1/14	0/2/13
	**overall *P***	0.644	0.744	0.936	0.289	0.547	**0.043**	0.074	0.159
Educatio-nal level (dietitians) †	Dietitian, BTS (n=4)	0/0/4	2/1/1	0/3/1	0/4/0	0/1/3	0/0/4	0/0/4	0/0/4
	Dietitian, pre-BSc (n=5)	0/0/5	0/1/4	0/0/5	0/4/1	1/2/2	1/1/3	0/0/5	0/0/5
	Dietitian, BSc (n=58)	0/5/53	4/24/30	2/9/47	3/21/34	1/16/41	1/10/47	1/0/57	2/1/55
	Dietitian, MSc (n=65)	0/5/60	5/23/37	1/13/51	3/27/35	2/15/48	0/12/53	2/1/62	2/5/58
	Dietitian, PhD (n=23)	0/1/22	2/11/10	0/3/20	3/11/9	2/2/19	1/0/22	0/0/23	0/1/22
	**overall *P***	0.880	0.143	0.150	0.129	0.268	**0.038**	0.961	0.848
Workplace †	Clinical setting (n=52)	0/8/44	6/28/18	2/14/36	4/25/23	3/12/37	0/8/44	0/0/52	2/4/46
	Community service (n=8)	0/1/7	0/5/3	0/1/7	2/3/3	0/2/6	2/0/6	0/1/7	0/1/7
	Industry (n=6)	0/0/6	0/1/5	0/0/6	0/1/5	0/1/5	0/2/4	0/0/6	0/0/6
	Academia/ Research (n=39)	0/2/37	2/16/21	1/6/32	3/13/23	2/6/31	1/7/31	1/0/38	1/4/34
	Freelancer (n=42)	0/0/42	4/9/29	0/5/37	1/19/22	1/13/28	1/5/36	0/0/42	0/0/42
	Other (n=17)	0/1/16	1/5/11	0/4/13	1/8/8	0/5/12	0/4/13	2/1/14	1/0/16
	**overall *P***	0.093	**0.049**	0.547	0.393	0.877	**0.013**	**0.006**	0.485

Years in practice as a dietitian†	0 – 4 (n=71)	0/7/64	4/24/43	1/14/56	4/33/34	2/18/51	2/12/57	3/0/68	4/3/64
	5 – 9 (n=33)	0/1/32	3/13/17	0/5/28	0/13/20	0/4/29	0/2/31	0/0/33	0/2/31
	10 – 19 (n=30)	0/3/27	2/12/16	0/6/24	3/13/14	3/5/22	1/5/24	0/0/30	0/0/30
	20 or more (n=21)	0/0/21	4/11/6	2/3/16	2/8/11	1/9/11	0/4/17	0/1/20	0/2/19
	**overall *P***	0.303	0.221	0.223	0.597	0.050	0.616	0.125	0.263
Total perceived knowledge	Poor (n=20)	0/5/15	0/12/8	0/7/13	1/15/4	0/10/10	2/6/12	0/0/20	0/1/19
	Average (n=83)	0/5/78	5/30/48	2/14/67	8/29/46	2/21/60	3/10/70	2/3/78	2/7/74
	Good (n=65)	0/4/61	7/22/36	1/9/55	4/27/34	4/12/49	1/11/53	1/0/64	2/3/60
	Excellent (n=11)	0/0/11	3/4/4	0/2/9	0/4/7	0/2/9	0/1/10	0/0/11	0/0/11
	**overall *P***	**0.021**	0.064	0.488	0.053	0.099	0.192	0.625	0.839
Total current Knowledge	1st quartile (n=45)	0/9/36	4/17/24	1/11/33	1/25/19	2/20/23	4/15/26	0/2/43	0/4/41
	2nd quartile (n=41)	0/3/38	2/17/22	1/9/31	4/22/15	1/11/29	1/4/36	0/1/40	0/2/39
	3rd quartile (n=51)	0/1/50	3/16/32	1/4/46	6/13/32	2/8/41	1/5/45	1/0/50	2/1/48
	4th quartile (n=42)	0/1/41	6/18/18	0/8/34	2/15/25	1/6/35	0/4/38	2/0/40	2/4/36
	**overall *P***	**0.004**	0.491	0.387	**0.020**	**0.021**	**0.001**	0.266	0.329

* Values are expressed as counts (n) of cases of “strongly don’t believe - don’t believe”/ “neutral (don’t know)” / “believe - strongly believe” for each variable category for the different health conditions; Numbers in parenthesis are counts (n) of cases per variable category and overall p depicts crosstabulation (chi-squared) analysis; †For European region n=175, for educational level and years in practice as a dietitian n=153, for workplace n=161; FBDGs: Food-Based Dietary Guidelines

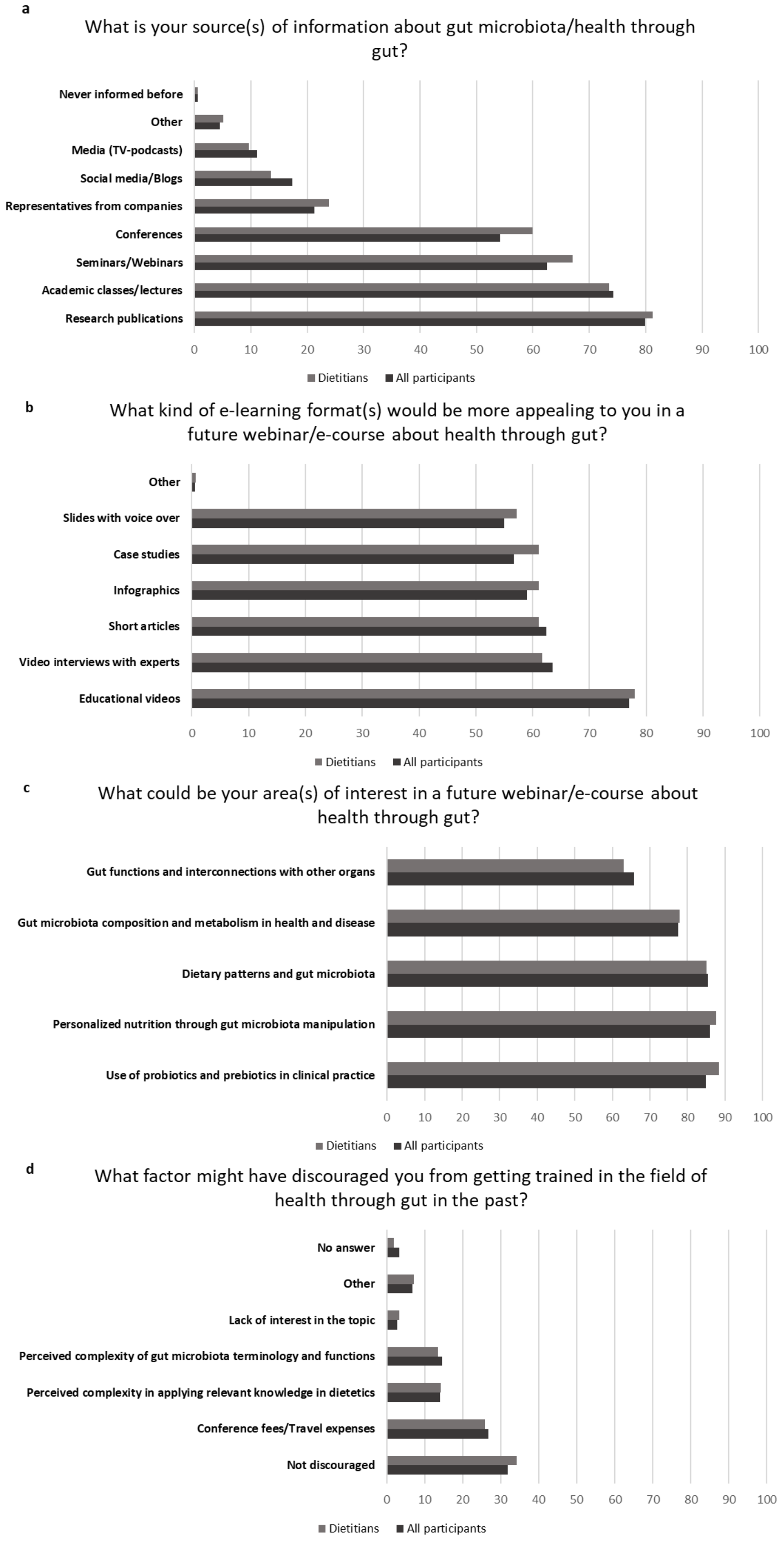

Figure S1 (a-d): Survey questions addressed to all participants (N=179) and dietitians (n=155) about sources of knowledge and interest in future initiatives regarding health through the gut; values are expressed as the percentage (%) of different answer options
